# Heterologous Expression of Recombinant Transglutaminase in *Bacillus subtilis* SCK6 with Optimized Signal Peptide and Codon, and Its Impact on Gelatin Properties

**DOI:** 10.4014/jmb.2002.02049

**Published:** 2020-04-23

**Authors:** Shiting Wang, Zhigang Yang, Zhenjiang Li, Yongqiang Tian

**Affiliations:** 1 Key Laboratory of Leather Chemistry and Engineering (Sichuan University), Ministry of Education, Chengdu 610065, P.R. China; 2 College of Biomass Science and Engineering, Sichuan University, Chengdu 610065, P.R. China; 3 Chengdu Jinkai Bioengineering Co., Ltd., Chengdu 611130, P.R. China

**Keywords:** MTG, heterologous expression, signal peptide optimization, *Bacillus subtilis* SCK6, enzymatic properties

## Abstract

Microbial transglutaminases (MTGs) are widely used in the food industry. In this study, the MTG gene of *Streptomyces* sp. TYQ1024 was cloned and expressed in a food-grade bacterial strain, *Bacillus subtilis* SCK6. Extracellular activity of the MTG after codon and signal peptide (SP Ync M) optimization was 20 times that of the pre-optimized enzyme. After purification, the molecular weight of the MTG was 38 kDa and the specific activity was 63.75 U/mg. The optimal temperature and pH for the recombinant MTG activity were 50°C and 8.0, respectively. MTG activity increased 1.42- fold in the presence of β-ME and 1.6-fold in the presence of DTT. Moreover, 18% sodium chloride still resulted in 83% enzyme activity, which showed good salt tolerance. Cross-linking gelatin with the MTG increased the strength of gelatin 1.67 times and increased the thermal denaturation temperature from 61.8 to 75.8°C. The MTG also significantly increased the strength and thermal stability of gelatin. These characteristics demonstrated the huge commercial potential of MTG, such as for applications in salted protein foods.

## Introduction

Transglutaminases (E.C. 2.3.2.13, TGases) are enzymes that catalyze cross-linking between the γ-carboxyamide group of a glutamine residue and the ε-amino group of a lysine residue [1]. TGases catalyze the formation of isopeptide bonds either within or between polypeptide chains and covalently incorporate polyamines into proteins with different primary amines, which eventually improve the solubility, thermal stability, water-holding capacity, and nutritional value of proteins [2]. TGases are widely distributed in various organisms, including humans, mammals, plants, and microorganisms [3]. In animals, TGases play an important role in various physiological processes [4] and neurodegenerative diseases [5], for which calcium (Ca^2+^) is required to expose cysteine residues in the active site domain [6]. Microbial TGases (MTGs) have unique advantages in industrial applications due to their Ca^2+^ independence, higher reaction rate, lower molecular weight, wide range of pH stability and broad substrate specificity [2]. MTG was first discovered in *S. mobaraensis* in 1989 and then gradually isolated from *Streptoverticillium* spp., *Streptomyces* spp., *Bacillus* spp., and some pathogenic strains, including *Candida albicans* [2, 5, 7, 8]. It has been widely used in the food industry to improve the functional properties and nutritional value of proteins. In recent years, MTG has shown application prospects in the cosmetic, textile, leather, pharmaceutical, and biomaterial sectors [2, 9].

*Streptomyces* MTG was first synthesized as an inactive zymogen and then processed to produce an active enzyme by removing N-terminal propeptide [10]. As production of active *Streptomyces* MTG causes cell death by cross-linking host proteins [11], MTG is usually expressed in a heterologous host in the form of pro-MTG. The proenzyme obtained by heterologous expression is converted to active MTG by coexpressing the protease [12] or by in vitro addition of activation protease [13]. To improve the yield of *Streptomyces* MTG, various hosts such as *Escherichia coli* [14], *Streptomyces lividans* [15], methylotrophic yeasts [16], *Corynebacterium glutamicum* [17], and *Yarrowia lipolytica* [3] have been investigated for heterologous expression.

Due to its nonpathogenic safety and high secretion properties, the expression host *Bacillus subtilis* is recommended by the US Food and Drug Administration and has been widely used as a host for heterologous protein expression. Compared with *E. coli*-based expression systems, the high secretion capacity of *Bacillus subtilis* provides better folding conditions and prevents the formation of inclusion bodies (IBs), which are biologically inactive [18]. Signal peptide plays a vital role in the translocation of secretory proteins across the plasma membrane. *B. subtilis* have four types of secretion pathways, most of which are the general secretion (Sec) pathway and the twin arginine translocation (Tat) pathway. The Sec-dependent secretory pathway is involved in secreting preprotein complexes with chaperone proteins, which bind to secreted transposases and facilitate transport across the plasma membrane. After removal of the signal peptide, the protein is released from the translocase and it refolds and passes through the cell wall [18-20]. Tian *et al.* [21] reported that the extracellular recombinant keratinase activity with the optimized signal peptide (SP_LipA_) was two times more than that of the wild type in *Bacillus subtilis* SCK6. Similarly, the recombinant amylase-producing strain with the best performing signal peptide (SP_pel_) yielded 68.4% more amylase than the natural strain [22]. Besides, codon optimization was also a commonly used method to increase the extracellular expression of proteins. Song Liu *et al.* [23] reported that the optimization of MTG gene based on the codon bias of *Streptomyces* increased MTG production by 73.6% in recombinant *S. lividans*.

To date, little research has been conducted on the heterologous expression of MTG in *Bacillus*. Since MTG is mainly applied in the food industry, *Bacillus subtilis* can secrete proteins outside the cell and is a better host for extracellular production of active-form TG. In this study, MTG with optimized codon and signal peptide was cloned and expressed in *B. subtilis* SCK6. Recombinant MTG was purified by ammonium sulfate precipitation and SP separation column. The active MTG was used to cross-link gelatin to provide evidence for the potential application of gelatin.

## Materials and Methods

### Bacterial Strains and Vectors

MTGase-producing bacterium *Streptomyces* sp. TYQ1024 (GeneBank Accession No. MN606211) was maintained in our laboratory. *Escherichia coli* DH5α (Vazyme, China) was used for vector construction. *Bacillus subtilis* SCK6 (BGSC 1A976) (Erm R, his, nprR2, nprE18, ΔaprA3, ΔeglS102, ΔbglT/bglSRV, lacA::PxylA-comK) was used as the expression host [24]. pMA0911 with Tat (Ywb N, Lip A, Amy X, Wap A) and Sec (Ync M, Npr E, Vpr, Yvg O) signal peptides was used as the expression vector.

### Cloning and Expression of MTG

Genomic DNA was extracted as described previously [25]. The DNA sequence encoding MTG was amplified using forward primer (5'-CGCGGATCCTCGCCACCGGCAGTGGCAGTGGCAGCG-3') with a BamHI recognition sequence and reverse primer (5'-CTAGCTAGCTCACGGCCAGCCCTGTGTCA-3') with a NheI recognition sequence (GeneBank Accession No. MN700931). The ligation recombinant plasmid was transformed into *E. coli* DH5α, and the transformants were inoculated on Luria-Bertani (LB) agar plate containing ampicillin (100 μg/ml) and incubated for 12 h at 37°C. The recombinant plasmid was amplified and verified by DNA sequencing (Sangon Biotech, Shanghai, China). One microliter of the recombinant plasmid was mixed with 100 μl of *B. subtilis* SCK6 competent cells and was allowed to grow in a shaking incubator (200 rpm) at 37°C for 90 min [26]. The competent cells were cultured overnight on LB agar plates with kanamycin (50 μg/ml) at 37°C. A positive clone was identified by PCR and shaken in a flask of LB liquid medium containing 50 mg/l kanamycin for 12 h. Then, 3% seed culture was inoculated into a fresh liquid fermentation medium with 50 mg/l kanamycin and allowed to grow in a shaking incubator (200 rpm) at 37°C for 60 h.

### Codon and Signal Peptide Optimization of MTG Gene for Expression in *Bacillus subtilis* SCK6

The coding region of MTG was chemically synthesized (Sangon Biotech, China) according to its preferred codon usage in the *Bacillus* strains. The codon-optimized MTG open reading frame (ORF) was cloned into the BamHI-NheI sites of pMA lipA, and the sequence of this synthesized fragment in pMA lipA-TGSO was confirmed by sequencing.

The recombinant plasmid was constructed and expressed as follows: pMA lipA-TG, pMA lipA-TGSO, pMA Ync M-TGSO, pMA Amy-TGSO, pMA wapA-TGSO, pMA Ywb N-TGSO, pMA Npr E-TGSO, pMA Vpr E-TGSO, pMA Yvg O-TGSO. The amplified MTG gene was ligated to pMA 0911 with different signal peptides to construct the expression vector.

### MTG Activity Assay

Transglutaminase activity was measured via hydroxamate assay [27] with few modifications. The culture supernatant (100 μl) was mixed with the substrate solution (1 ml) with 30 mM CBZGln-Gly, 0.2 M Tris-HCl buffer (pH 6.0), 10 mM glutathione and 100 mM hydroxylamine, and allowed to react at 37 °C for 10 min. Then, the reaction was stopped by adding ferric chloride trichloroacetic acid reagent. The TG activity was determined at the absorbance of 525 nm. The calibration curve was prepared with L-glutamic acid γ-monohydroxamate. One unit of enzyme activity was defined as the amount of enzyme required to produce 1 μmol of hydroxamic acid per minute at 37 °C.

### Purification of Recombinant MTG

The fermentation broth was centrifuged at 7,000 ×g for 10 min to remove the precipitate. Ammonium sulfate was added to a final saturation of 50% (25°C) of the pellet supernatant and centrifuged at 7,000 ×g for 20 min to remove the pellet. Ammonium sulfate was again added to a final saturation of 80% (25°C), and the supernatant was centrifuged at 7,000 ×g for 20 min to obtain a precipitate. The precipitate was dissolved in 20 mM sodium phosphate buffer (PB; pH 5.8) and centrifuged to remove the pellet. The supernatant was chromatographed on a SP Sepharose Fast Flow column previously equilibrated with buffer A (PB; pH 5.8). Adsorbed MTG was eluted with a linear gradient of sodium chloride (NaCl; 0–1 M). The active fractions were combined and stored at -20°C. SDS-PAGE was performed using 12.5% separating polyacrylamide gel for establishing the purity and the molecular mass of the MTG.

### Biochemical Characterization of MTG

To determine the optimal pH of MTG, substrate solutions of different pH were prepared. We used 100 mM citrate buffer in the pH range 3–5, 100 mM phosphate buffer in the pH range 6.0–8.0, Tris-HCl buffer in the pH range 8.0–10.0, and glycine/NaOH buffer at pH 11.0. To determine the effect of pH on the stability of MTG, the enzyme was preincubated with the corresponding buffer (1:1 ratio) at 37°C for different durations, and the residual enzyme activity was measured.

The optimal temperature of MTG was measured at 10°C, 20°C, 30°C, 40°C, 50°C, 60°C, and 70°C (pH 7.0). To determine the effect of temperature on MTG stability, the enzyme solution was incubated at 20°C to 60°C. Samples were selected every hour and the residual enzymatic activity was measured.

The effect of different chemical substances such as dimethyl sulfoxide (DMSO), phenylmethylsulphonyl fuoride (PMSF), ethylene diamine tetraacetic acid (EDTA), sodium dodecyl sulfate (SDS), Tween-20, β- Mercaptoethanol (β-ME), a few metal ions, and other organic solvents on MTG was studied. All chemicals were preincubated at 37°C for 30 min. Then the residual enzymatic activity was measured after mixing with purified TG at 37°C for 10 min. The effect of NaCl concentrations on MTG was measured at 2%, 4%, 6%, 8%, 10%, 12%, and 14% at 37°C for 10 min.

The Michaelis-Menten equation was used to determine the kinetic parameters of MTG, which were examined under the substrate of CBZ-Gln-Gly from 5 to 30 mmol/l (pH 6.0). The values of Michaelis constant (K_m_) and maximum velocity (V_max_) of MTG were calculated from the Lineweaver-Burk plot.

### Effect of MTG on Gelatin Properties

**Measurement of gelling time and gel strength.** Gelatin (240–270 Bloom) was purchased from Sangon Biotech (China). MTG was obtained from *Bacillus subtilis* SCK6 (pMA Ync M-TGSO). Gelatin in different concentrations (6%, 8%, 10%, 12%, and 14%) was weighed and dissolved in distilled water at 50°C. To this, MTG (0.1, 0.2, and 0.5 U/ml) was added and mixed. The gelatin was reacted at 42°C and the length of time until the solution could be tilted 90° with no liquid flowing out, was measured. Note: The meaning of this sentence is not clear.

The gel strength before and after MTG modification was measured using a texture analyzer (TA-XT Plus Texture Analyser, Lotun Science Co., Ltd., China). Gels were compressed at a rate of 1 mm/s with P/10R probe until 4 mm of penetration was reached. The maximum force for gelatin was considered as gel strength.

**Differential scanning calorimetry (DSC).** Thermal properties of gelatin were measured by DSC (204 F1, NETZSCH, Germany). After the gel was freeze-dried, samples (3–5 mg) were sealed in an aluminum pan to measure the DSC value, and then heated from 10°C to 150°C at a rate of 10°C/min. Meanwhile, an empty aluminum crucible was used for comparison.

**Fourier transform infrared spectroscopy (FTIR).** FTIR spectra of gel were determined using FTIR spectrometer (Nicolet IS 10, Thermo Scientific, USA). The hydrogel, before and after modification, was freeze- dried to obtain a dry sponge. With 5–10 mg of dried gel, we used the potassium bromide grinding and tableting method to make a circular sheet. The round tablet was fixed to the sample holder of the infrared spectrometer. Infrared scanning was performed in the spectral range of 4,000~500 cm^-1^, and the infrared spectra of gels before and after modification were obtained.

## Results and Discussion

### Cloning and Expression of MTG

**Fig. 1:**
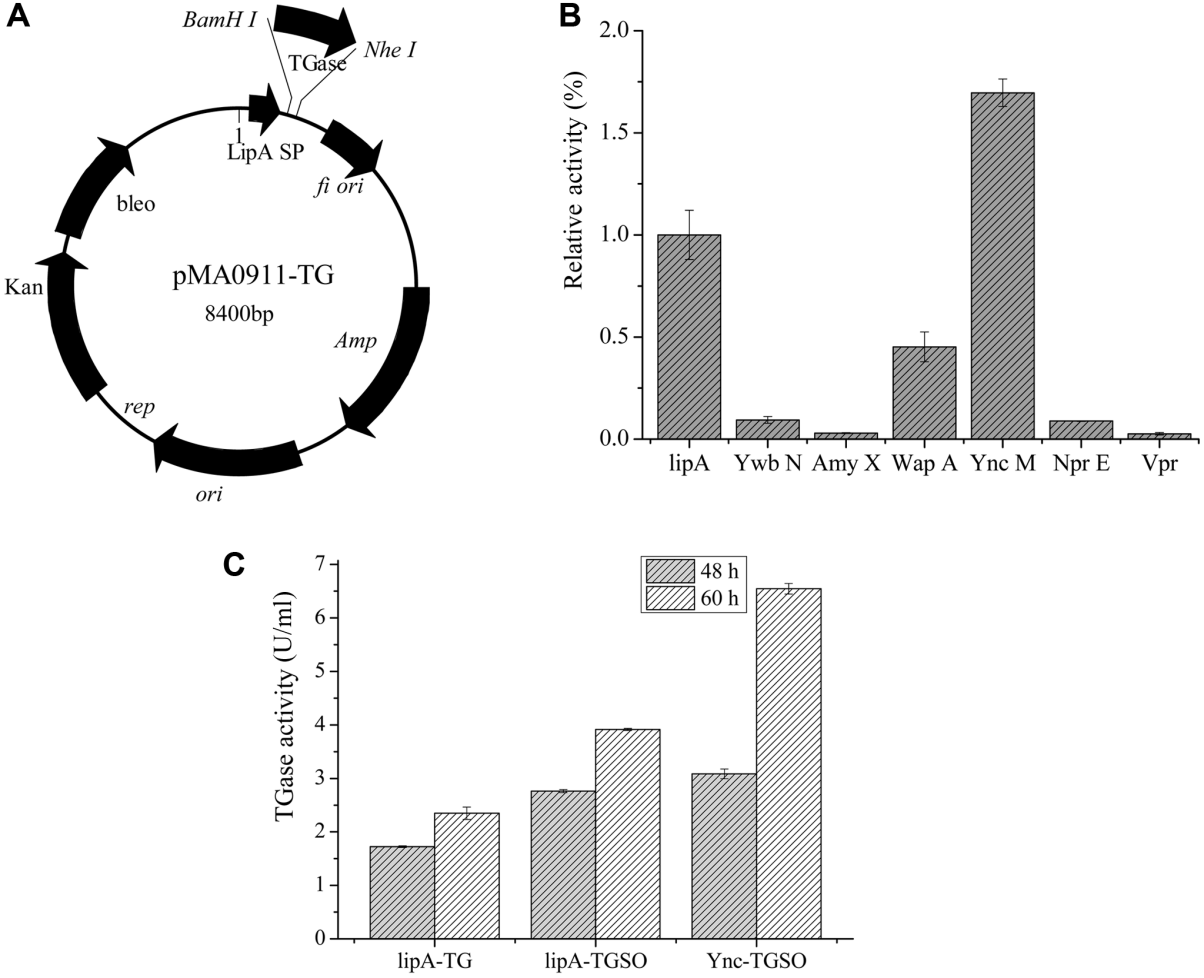
Heterologous expression of transglutaminase in *Bacillus subtilis* SCK6 with codon and signal peptide optimization. (**A**) Expression of recombinant vector pMA lipA-TG. The signal peptide lipA is above BamHI ; the pro-TGase gene is inserted between the BamHI and NheI sites; (**B**) TGase activity assay of the different signal peptide. (**C**) The recombinant pMA lipA-TG; pMA lipA-TGSO; pMA YncM-TGSO were inoculated into 50 ml of medium (containing 50 μg/ ml Kan) and cultured at 37°C and 200 rpm for 48 h and 60 h, respectively.

The *Streptomyces* MTG ORF consists of sequences that code for a secretory signal peptide, a pro-peptide gene, and a mature MTG gene. The upstream sequences of the ORF contain a putative promoter and the downstream sequences contain a putative terminator [28]. *Bacillus subtilis* is one of the most widely used hosts for protein production due to high secretion, excellent safety, clear genetic background, and well-developed fermentation technology [21]. In order to express the MTG in *B. subtilis* SCK6, the MTG gene (pro-mature, 1,149 bp) amplified from *Streptomyces* sp. TYQ1024 genome was inserted downstream of a secretion signal lipA in pMA lipA vectors which resulted in the plasmid pMA lipA-TG (Fig. 1A). The MTG activity of the culture supernatant arrived at 2.34 U/ml, after shake-flask fermentation for 60 h (Fig. 1C).

### Codon and Signal Peptide Optimization of MTG Gene

To improve MTG expression in *Bacillus subtilis* SCK6, the codon of MTG was optimized for expression in *Bacillus* and was chemically synthesized by Sangon Biotech. The codon-optimized MTG ORF was then cloned into BamHI-NheI sites of pMA lipA, which yielded pMA lipA-TGSO. pMA lipA-TGSO expression in *B. subtilis* SCK6 yielded the highest MTG (3.92 U/ml) at 60 h, which was 67.5% more than the control strain produced by pMA lipA-TG expression in *B. subtilis* SCK6 at 60 h (2.34 U/ml) (Fig. 1C). This is consistent with the expression of MTG in *Streptomyces lividans* when certain rare codons were replaced with preferred codons and resulted in 73.6% enhanced MTG production [23]. The low level of transfer RNA molecules prevents MTG expression when rare codon charged transfer RNA molecules are much lower than abundant codons [29]. The sequence optimization resulted in 67.5% more MTG production in *B. subtilis* SCK6.

To improve the extracellular expression of MTG, three signal peptides (SP_YncM_, SP_NprE_, and SP_Vpr_) from the Sec pathway and four signal peptides (SP_YwbN_, SP_LipA_, SP_AmyX_, and SP_WapA_) from the Tat pathway were selected to construct recombinant MTG plasmid. The recombinant strains with different signal peptides were incubated in the fermentation liquid medium to determine extracellular MTG activity for 60 h. Among the seven signal peptides, SP_YncM_ exhibited a secretion efficiency of 6.7 U/ml, which was 69% more than SP_LipA_. Meanwhile, the other signal peptides (SP_WapA_ and SP_YwbN_) showed a lower secretion efficiency (Fig. 1B). These findings confirm that Sec-pathway signal peptide SP_YncMA_ directs efficient MTG secretion.

### Purification and Biochemical Characterization of MTG

**Fig. 2:**
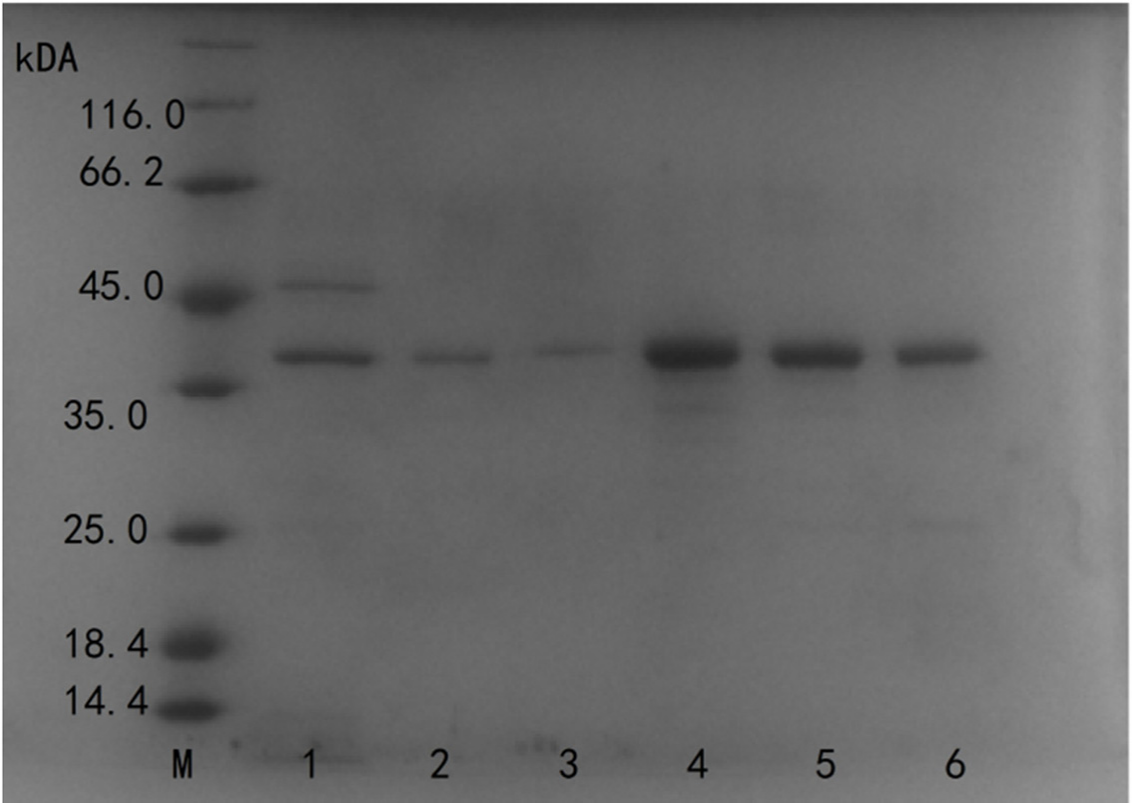
SDS-PAGE analysis of purified TGase. M Protein Marker in different molecular weight size; lane 1 was fermentation supernatant; lanes 2, 3, 4, 5, 6 were purified active TGase by SP FF.

*Bacillus subtilis* SCK6 expressed MTG as a soluble protein when incubated at 37°C for 60 h. The fermentation supernate was initially precipitated with 50%–80% saturated ammonium sulfate. Then, the precipitate was dissolved in 20 mM PB at pH 5.8 (buffer A) and was subjected to SP Sepharose Fast Flow. The purified, mature MTG had a molecular mass of 38 kDa (SDS-PAGE, lane 2 to 6; Fig. 2). The purified enzyme exhibited a specific activity of 63.75 U/mg of protein with 8.53-fold purification and 23.36% recovery compared to the fermentation supernatant (Table 1). Compared with the commonly used *E. coli* expression system, *Bacillus subtilis* does not form inclusion bodies and endotoxin. Moreover, the extracellular expression of MTGase effectively avoidscomplicated operations, such as cell disruption, dilution and renaturation, which facilitates the separation and purification of enzymes. Yokoyama K expressed MTGase in *E. coli* JM 109 reached enzyme activity of 1.35 U/ml with refolding and renaturation in vitro [30]. Yi-Sin Lin expressed mtgA from *Streptomyces platensis* in *Streptomyces lividans* JT46/pAE053 with 2.2 U/ml MTGase activity [31]. In this study, the MTGase expressed in *B.subtilis* SCK6 exhibited a secretion efficiency of 6.7 U/ml, showed a large yield advantage and effectively reduced the cost of industrial production.

**Fig. 3:**
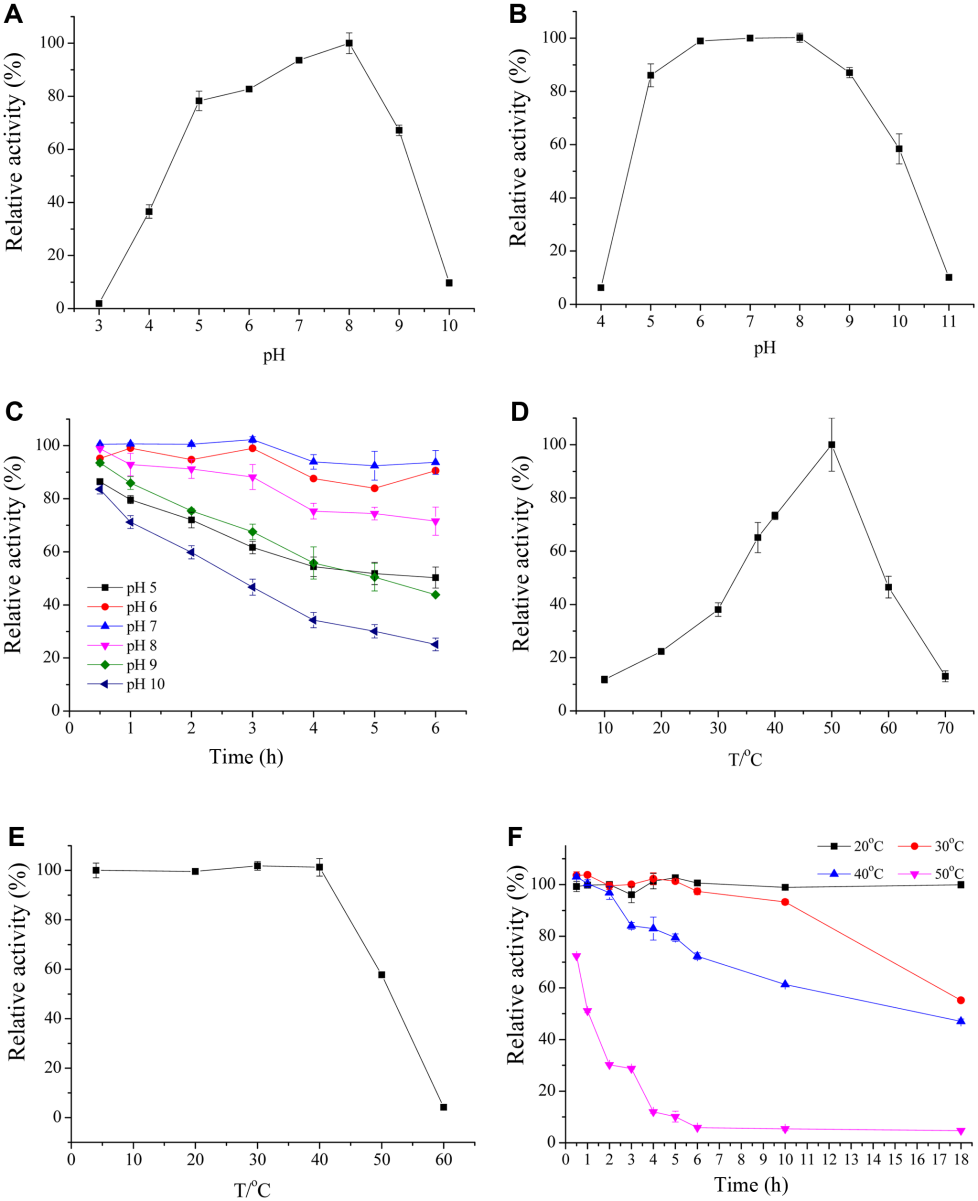
Effects of pH and temperature on the activity and stability of MTGase. (**A**) Activity of MTGase at different pH; (**B**) stability and pH at 30 min; (**C**) stability and pH at 30 min to 6 h; (**D**) activity of MTGase at different temperatures; (**E**) stability and temperature at 30 min; (**F**) stability and temperature at 30 min to 18 h.

The pH of the reaction mixture affects the conformation and configuration of the active and catalytic sites of the enzyme as well as the net charge of the protein in its hydrogen bonding pattern [32]. Therefore, we investigated the effect of pH on MTG activity (pH 3.0–10.0; Fig. 3A). The optimum pH of MTG from recombinant SCK6 was in the range of 7 to 8, which is consistent with that observed for MTG from *Streptomyces* [33]. MTG was stable (residual activity > 70%) over a wide range of pH (5.0–9.0) and maintained high stability (residual activity > 80%) in the pH range of 6–7 after 6 h (Figs. 3B and 3C).

We further investigated the effect of temperature (10–70°C) on the enzyme activity. MTG activity was maximum at 50°C (Fig. 3D). The optimal temperature of recombinant MTG in this study was similar to that of MTG isolated from *Streptoverticillium mobarense*, whereas, it was higher than that of MTG isolated from *Streptomyces hygroscopicus* (37–45°C) [8]. MTG exhibited stable activity in the temperature range of 0 to 40 °C at 30 min, and the activity dropped at temperatures above 50°C (Fig. 3E). There was no significant loss in enzyme activity at 20 °C; however, half of the MTG activity was lost at 30°C and 40°C at 18 h (Fig. 3F).

The relative activity of MTG was measured in the presence of different metal ions at 5 mM concentration (Table 2). Cr^3+^, Fe^2+^, Fe^3+^, Cu^2+^, Zn^2+^, and CrO_4_^2-^ strongly inhibited the enzyme activity, while Ni^+^ and Pb^2+^ slightly inhibited the activity. Ca^2+^, Mn^2+^, Mg^2+^, Li+, Ba^2+^, Co^2+^, and K^+^ did notinhibit MTGaseactivity. The reductants DL- Dithiothreitol (DTT, 142.42 ± 1.84%, 5%) and β-Mercaptoethanol (β-ME, 160.18 ± 1.5, 5 mm) enhanced MTG activity, while PMSF and SDS inhibited MTG activity. H_2_O_2_ at 1% concentration completely inhibited MTG activity (1.43 ± 1%). In addition, when the concentration was reduced to 0.5%, the inhibitory effect on H_2_O_2_ weakened (35.86 ± 0.45%). Interestingly, 10% acetone completely inhibited MTG activity, and the inhibition weakened as the concentration reduced to 5%. Heavy metals such as Cu^2+^, Fe^3+^, Zn^2+^, and Cr^3+^ will bind the thiol group of the single cysteine residues to inhibit MTGase activity, which supports the theory that cysteine residues are the active site of MTGase [2]. Among them, Ca^2+^ has no obvious effect on enzyme activity, showing that the microbial source TGase is Ca^2+^ independent. There was no metal ion involved in the active center of the MTGase. Therefore, the metal ions such as Ca^2+^, Mg^2+^, Li+, Ba^2+^, and K^+^ have little effect on the activity of MTGase. The antioxidants β-ME and DTT effectively promote the MTG enzyme activity. This may be that β-ME and DTT can protect the thiol group of the TGase active center from being oxidized, and meanwhile it can also reduce the cross- linked bond to a disulfide bond [34].[Table T3]

**Fig. 4:**
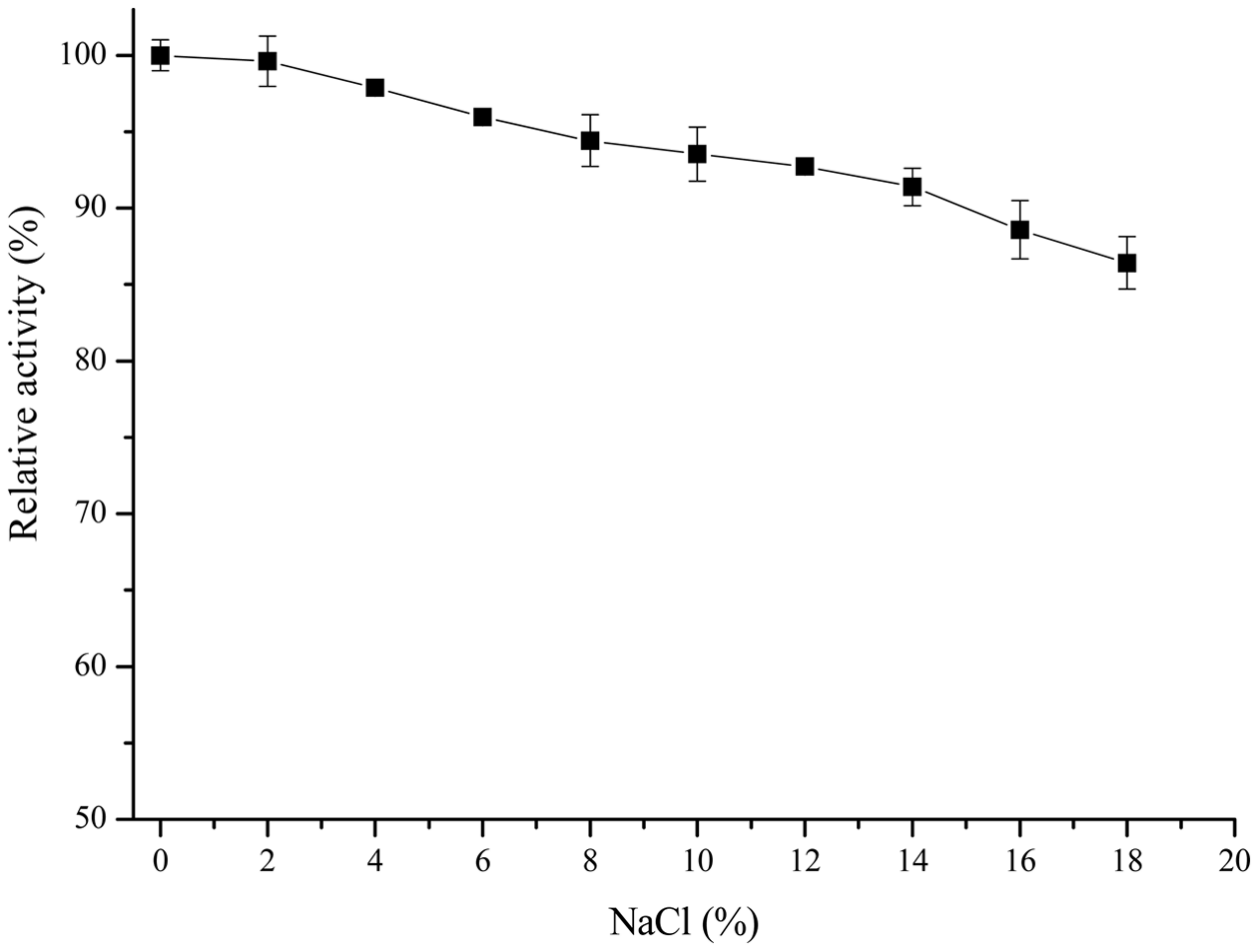
TGase activities in different concentrations of NaCl.

MTG activity in the presence of NaCl at different concentrations revealed NaCl tolerance of MTG (Fig. 4). MTG exhibited about 80% activity in the presence of NaCl at 18% concentration (w/v). This finding indicates it advantage over normal MTGs, which are unstable in salt. Salt is commonly used in food processing, and people in the Far East often use salt to marinate foods, especially protein foods such as pork, fish and sausages. These foods with salt have a longer storage time and a special flavor. In addition, Carlos Cardoso found that MTGase and salt have a synergistic effect on the production of high-quality gels from farmed sea bass, especially the enhancement of gel strength [35]. Therefore, this salt tolerance indicates the great potential of MTGase in high-salt food applications.

**Fig. 5:**
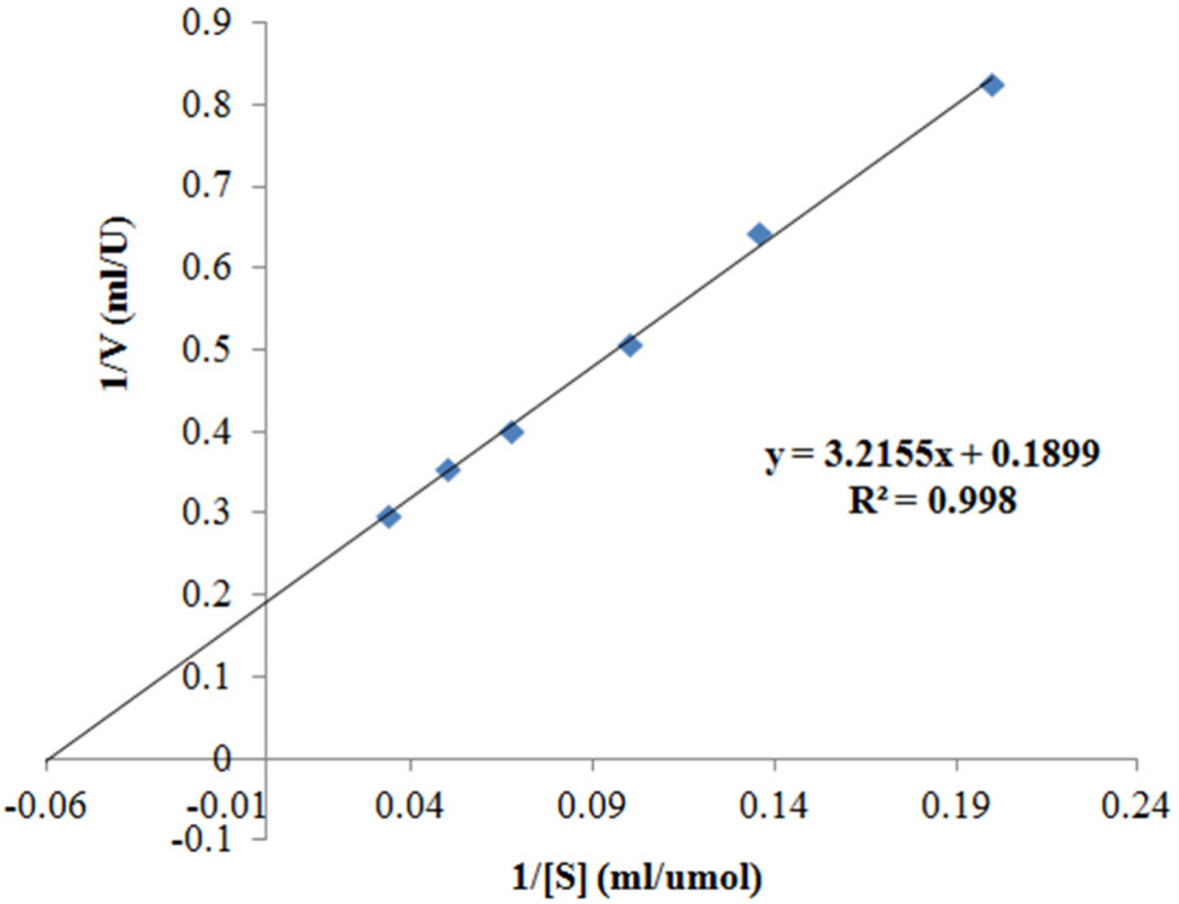
Kinetic parameters of MTGase from SCK6.

The kinetic parameters, K_m_ and V_max_, were determined as shown in the plot (Fig. 5). The Michaelis-Menten constant (K_m_) was 16.93 μM/ml, whereas V_max_ was 5.27 U/min. A low value of K_m_ indicates that the substrate is held tightly and the enzyme will achieve maximum velocity at low substrate concentration.

### Effect of MTG on Gelatin Properties

**Fig. 6:**
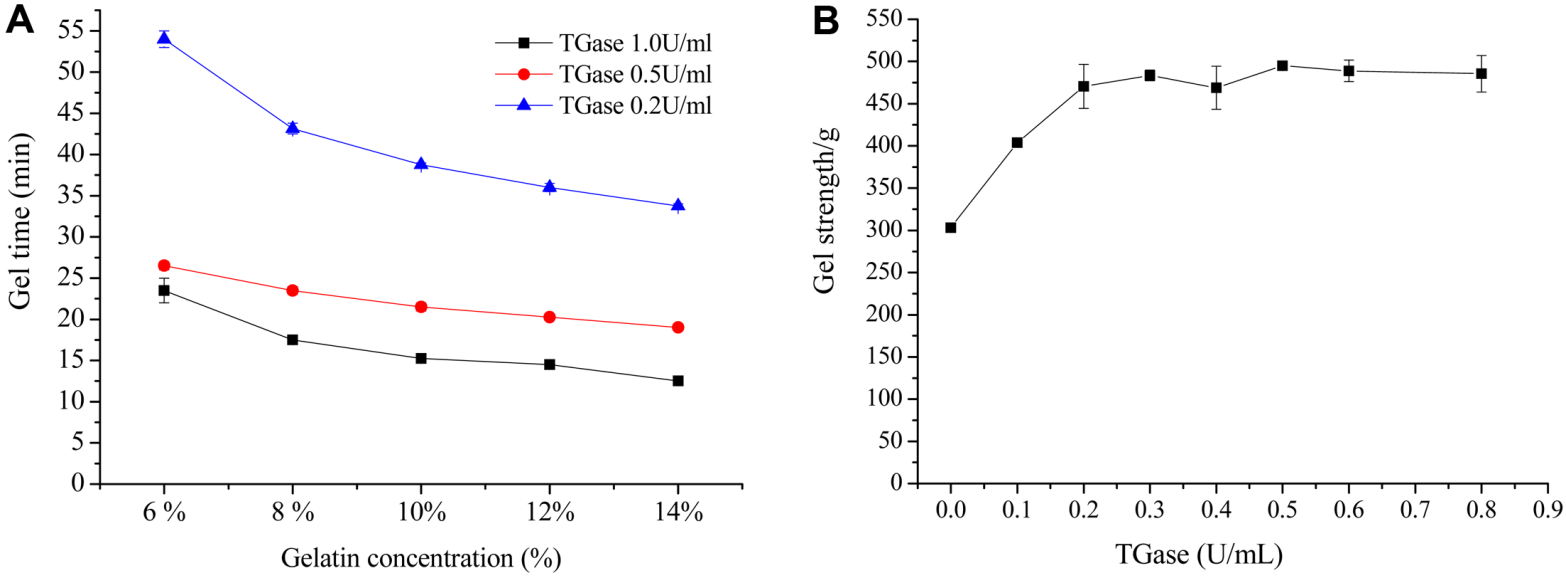
(A) Influence of gelatin and TGase concentration on gelling time; (B) Effects of different concentrations of MTG on gel strength of gelatin gels.

**Gelling time and gel strength.** The relationship between gelatin and MTG concentrations and the time required to form a gel is shown in Fig. 6A. With increase in gelatin concentration from 6% to 14%, the gelling time had a significant reduction from 54 min to 33 min with addition of 0.2 U/ml MTG. Similarly, with increase in MTG concentration, gelling time decreased significantly. MTG at 1 U/ml completely cross-linked 14% gelatin in 11 min. The gelatin at high concentration provided more cross-linking sites for MTG, which made the reaction proceed smoothly. In general, gelatin solution can be physically cross-linked between gelatin molecules to form a gel without adding any cross-linking agent when the temperature is below 29°C and dissolved into aqueous solution when temperatures are above 30°C [36]. Interestingly, MTG-cross-linked hydrogel did not hydrolyze at high temperatures, which expands the application of hydrogels.

Gel strength is one of the major physical properties of gelatin and is considered as a stiffness factor to predict physical properties [37]. The gel strength of MTG-modified gelatin first increased and then stabilized at 490 g with 0.3 U/ml of MTG, which was about 1.65-fold that of the control gel (Fig. 6B). The network structure of gel showed an obvious effect on its texture properties and closely entangled gels showed greater gel strength. MTG catalyzes the formation of covalent bonds, then propitiates a stronger and more stable gel network [38]. This indicated that the physical properties of the gel improved by MTG modification.

**Fig. 7:**
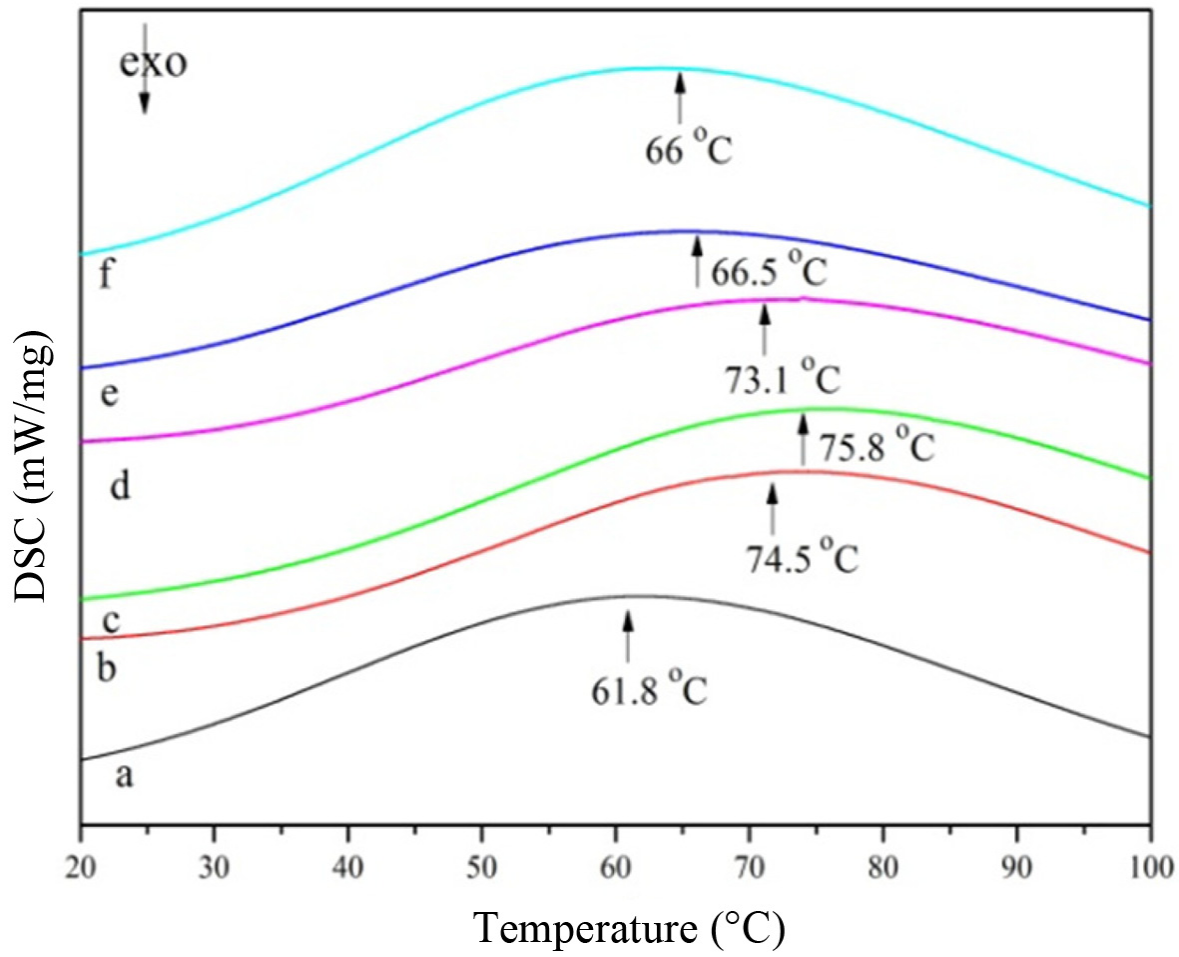
Effects of different concentrations of MTG on thermal stability of gelatin. a: 0 U/ml; b: 0.1 U/ml; c: 0.2 U/ml; d: 0.3 U/ml; e: 0.4 U/ml; f: 0.5 U/ml.

**Differential scanning calorimetry (DSC).** The thermal properties of gelatin were measured by differential scanning calorimetry (DSC), which is an effective method to provide the thermodynamic data (Tm). MTG provided to gelatin gels increased their thermal denaturation temperature (Fig. 7). The thermal denaturation temperature of modified gelatin gels increased from 61.8 to 75.8°C with increase in MTG concentration from 0 to 0.3 U/ml. This is consistent with the findings of Liu Fei *et al.* who found that MTG improved the mechanical and thermal properties of cross-linked gelatin film [39]. However, as the MTG enzyme concentration increased to 0.5 U/ml, the thermal denaturation temperature of the modified gelatin decreased from 75.8 to 66°C. This may be due to the cross-linking of gelatin under the catalysis of MTG, which formed gel networks with higher molecular weight and made the gelatin tangles tighter and the voids smaller [40]. Therefore, the bonds of network structure between gelatin in the presence of MTG are more difficult to break. At MTG concentrations above 0.3 U/ml, the local reaction inside the gelatin may cause a steric hindrance effect, which is not conducive to improving the thermal stability.

**Fig. 8:**
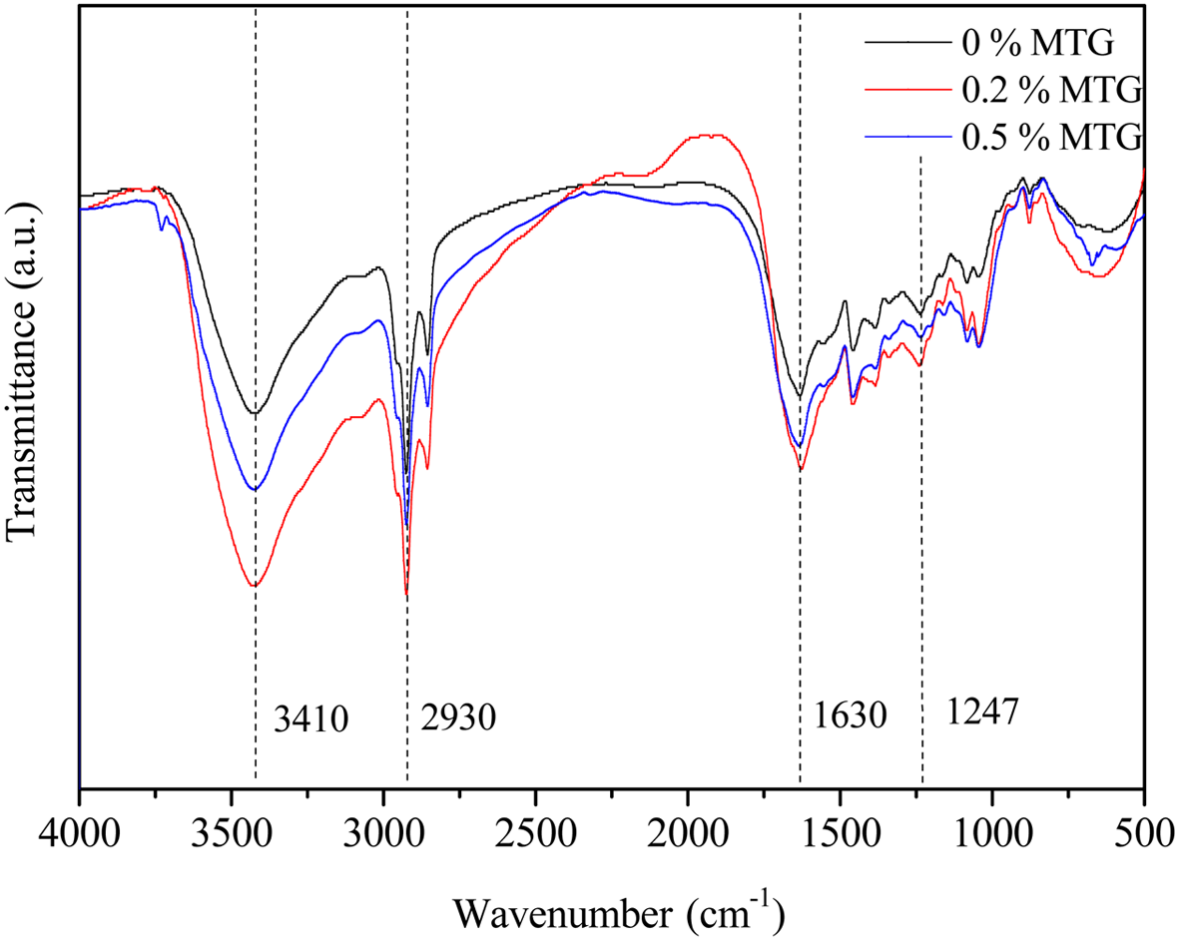
FI-IR spectrum of crosslinked gelatin gels under different concentrations of MTG.

**Fourier transformation infrared (FTIR) spectroscopy.** FTIR spectra of cross-linked gels at different concentrations of MTG are shown in Fig. 8. The gelatin spectral distribution showed characteristic absorption bands. The absorption band near 3,410 cm^-1^ corresponds to amide A and may be assigned to the vibration of OH and NH groups [41]. The absorption band near 2,930 cm^-1^ corresponds to the amide B and may be assigned to the vibration of =C-H and -NH groups [42]. The characteristic absorption bands of amide I (C=O and CN stretching vibration), amide II (NH and CN groups vibrations), and amide III (vibrations of NH and CN groups) were observed at 1,630, 1,547, and 1,240 cm^-1^, respectively [43]. The absorption peaks did not shift significantly when gelatin was modified with MTG at different concentrations. FTIR spectra showed that the band strength of amide A and amide I enhanced, which was due to the addition of MTG that promoted the cross-linking of gelatin (to form more isopeptide bonds) and increased the number of NH bonds.

In this study, codon and signal peptide optimization improved the extracellular expression of MTG in *B. subtilis* SCK6 (Ync M-TGSO) which showed the highest extracellular MTG activity (6.7 U/ml, 2.86-fold). The enzyme showed maximum activity at pH 8 and 50°C. The recombinant MTG showed tolerance to sodium chloride and organic solvents. The strength and thermal stability of gelatin significantly increased with MTG cross-linking. This is the first study to report the secretion of MTG in *Bacillus subtilis* SCK6, with a final recovery of 63.75 U/mg without any chemical inducer. In conclusion, this study provides a new strategy for the efficient production of MTG that could be used in salt-containing foods.

## Figures and Tables

**Table 1:** Purification of TGase.

Purification step	Total activity (U)	Total protein (mg)	Specific activity (U/mg)	Purification fold	Yield (%)
Supernatant	3186.46	426.47	7.47	1	100
Ammonium sulfate	2556.77	100.24	25.51	3.41	80.20
SP FF	744.31	11.68	63.75	8.53	23.36

**Table 2:** Effects of metal ions on TGase activity.

Metal ions	Concentration	Residual activity (%)
Control	-	100 ± 2.33
Ca^2+^	5 mM	101.32 ± 0.14
Cu^2+^	5 mM	35.76 ± 0.071
Mn^2+^	5 mM	96.23 ± 0.28
Mg^2+^	5 mM	101.04 ± 2.05
Fe^2+^	5 mM	11.98 ± 0.35
Zn^2+^	5 mM	32.83 ± 0.42
Fe^3+^	5 mM	14.90 ± 0.85
Ni^+^	5 mM	64.91 ± 0.84
Li^+^	5 mM	94.13 ± 2.33
Ba^2+^	5 mM	101.56 ± 1.13
Co^2+^	5 mM	91.01 ± 1.77
Cr^3+^	5 mM	5.53 ± 0.92
CrO_4_^2-^	5 mM	25.98 ± 1.63
Pb^2+^	5 mM	69.44 ± 2.48
K^+^	5 mM	102.00 ± 2.22

**Table 3 T3:** Effects of chemicals and various surfactants on TGase activity.

Chemicals	Concentration	Residual activity (%)	Concentration	Residual activity (%)
Control	-	100 ± 1.5	-	100 ± 2.4
Tween 20	10%	75.64 ± 5.06	5%	93.26 ± 4.34
Tween 80	10%	67.58 ± 23.83	5%	97.39 ± 2.88
Glycerin	10%	99.02 ± 6.42	5%	103.91 ± 0.96
Acetone	10%	5.89 ± 0.15	5%	89.72 ± 1.89
Ethanol	10%	90.96 ± 2.88	5%	101.96 ± 0.95
Benzene	10%	96.46 ± 0.46	5%	101.14 ± 1.13
DMSO	10%	90.37 ± 1.56	5%	99.41 ± 0.47
Isopropanol	10%	91.75 ± 2.92	5%	97.64 ± 0.23
Formamide	10%	92.53 ± 7.67	5%	97.98 ± 2.49
Hexane	10%	96.46 ± 0.31	5%	99.65 ± 0.31
Urea	0.5 M	86.15 ± 1.56	0.25 M	90.93 ± 1.84
GNHCL	0.5 M	89.88 ± 1.9 1	0.25 M	98.92 ± 0. 57
SDS	0.1%	7.641 ± 5.66	0.05 %	2.9 ± 3.25
β-ME	10 mM	126.55 ± 2.2	5 mM	142.42 ± 1.84
PMSF	10 mM	4.85 ± 5.60	5 mM	7.46 ± 0. 50
DDT	10 mM	150.57 ± 1.85	5 mM	160.18 ± 1.5
Tritonx-100	5%	70.95 ± 3.75	2.5%	92.99 ± 2.7
H_2_O_2_	1%	11.43 ± 1	0.5%	35.86 ± 0.45
EGTA	10 mM	99.43 ± 2.5	10 mM	101.17 ± 1.1
EDTA	10 mM	91.24 ± 1.5	10 mM	96.56 ± 2.25
